# Supplementary figures and images for: Structural implications of BK polyomavirus sequence variations in the major viral capsid protein Vp1 and large T-antigen: a computational study
Source: mSphere. 2024 Mar 19;9(4):e00799-23. doi: 10.1128/msphere.00799-23 (PMC11036806; doi:10.1128/msphere.00799-23)

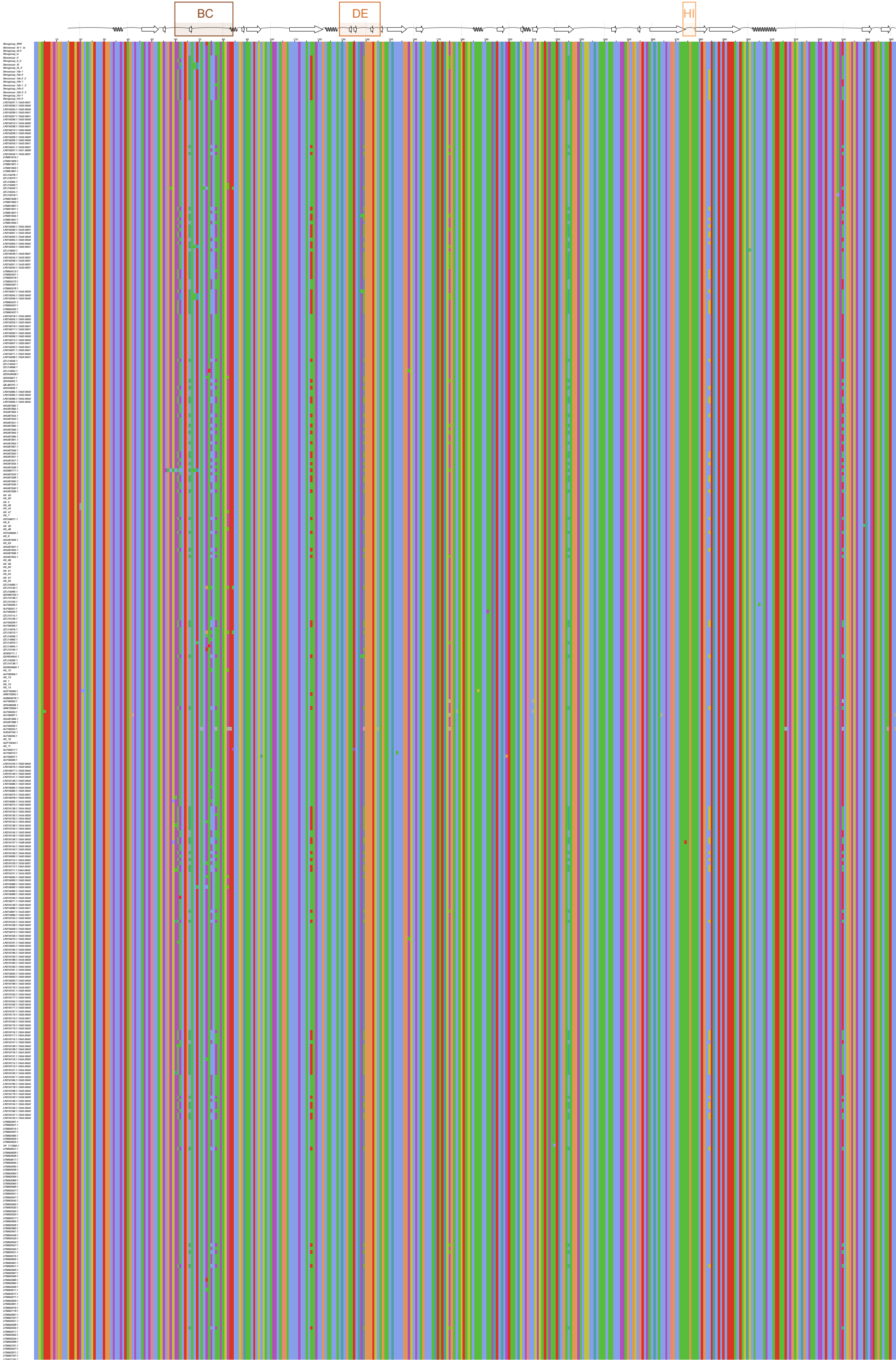

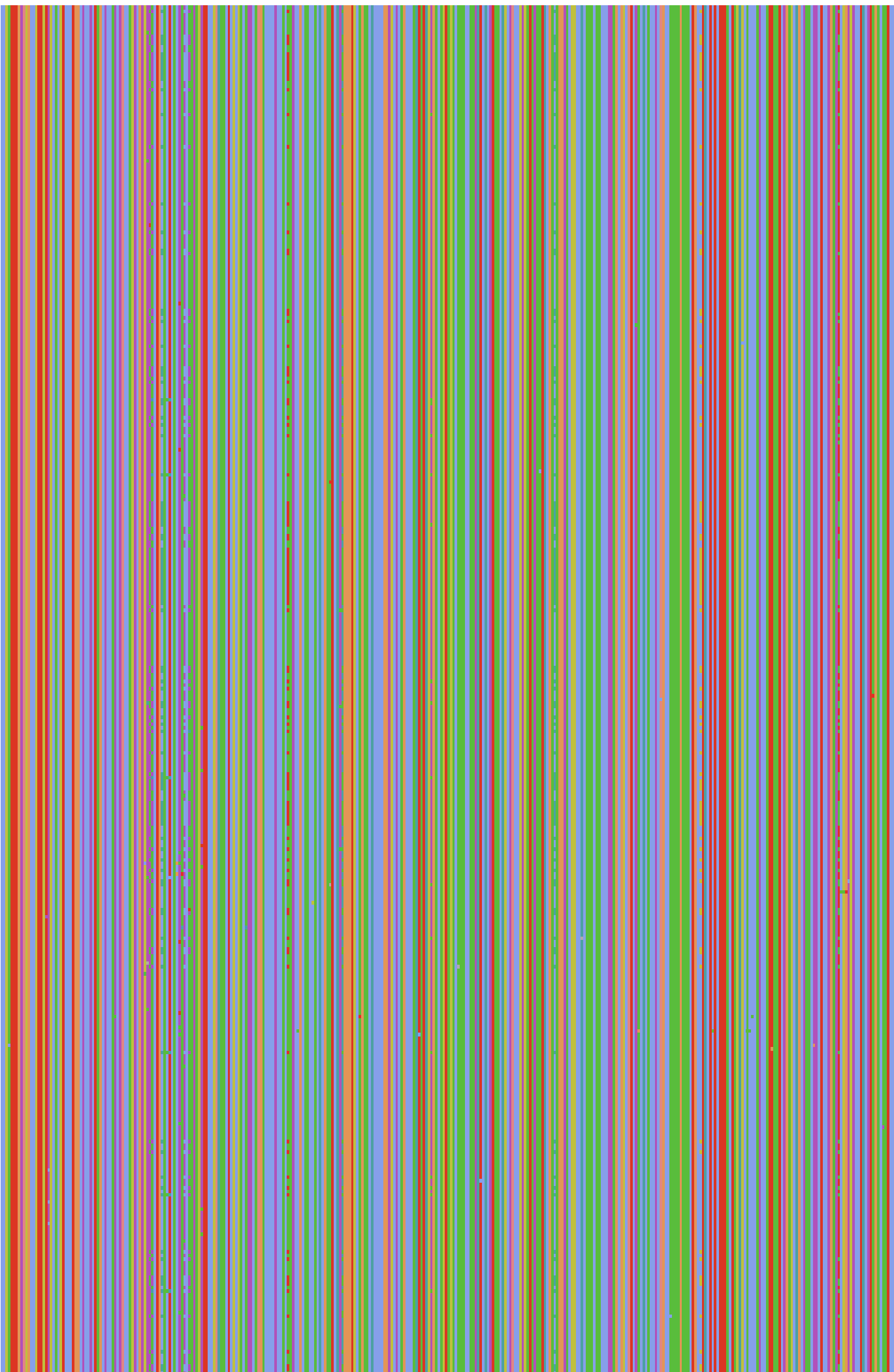

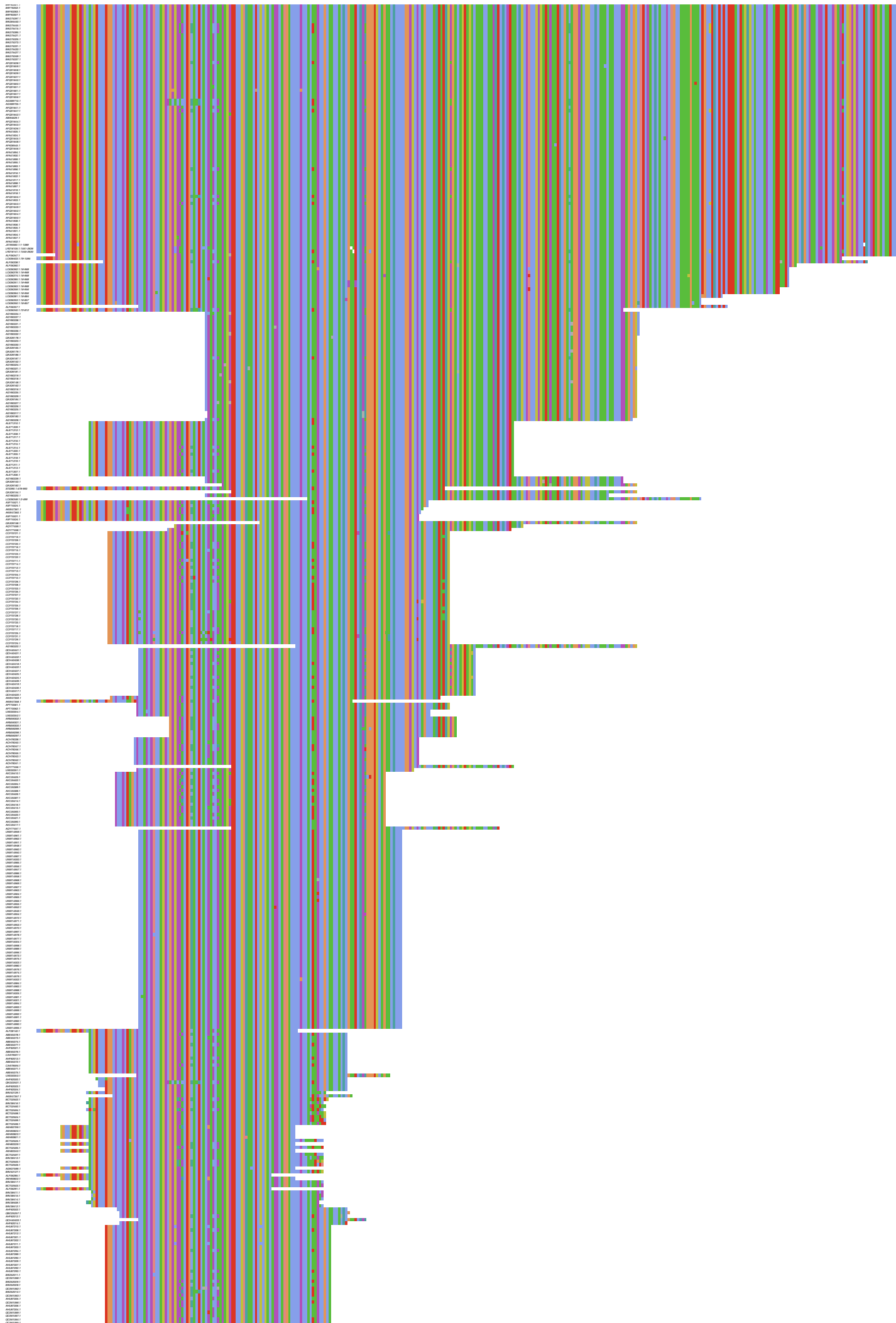

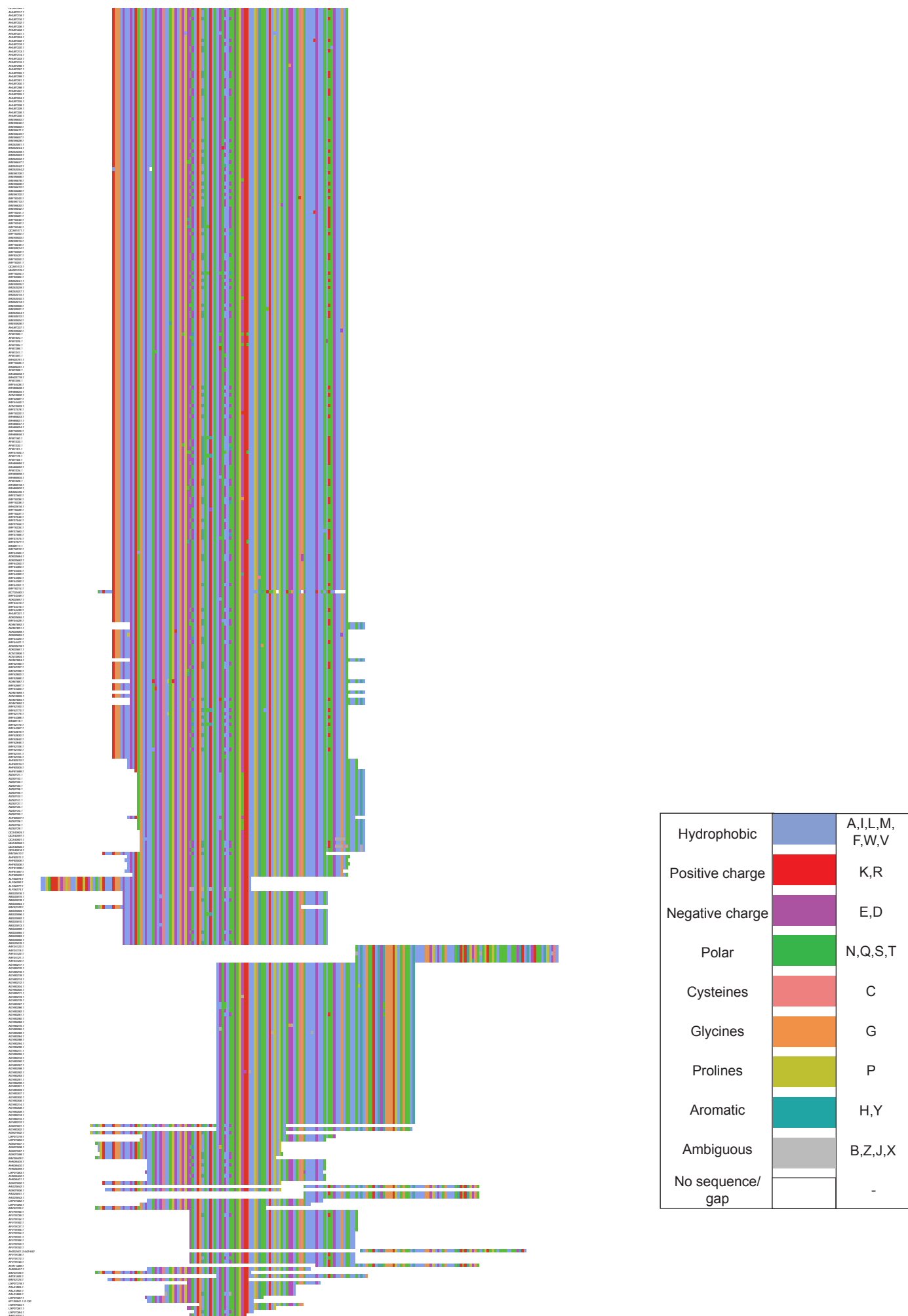

**Supplementary Figure 1. Vp1 alignment.**

Rendered in JalView [1] following the Clustal color scheme [2].

Supplement: Fig. S1 — Vp1 sequences aligned. [file msphere.00799-23-s0001.pdf]
