# Supplementary material for: Structural implications of BK polyomavirus sequence variations in the major viral capsid protein Vp1 and large T-antigen: a computational study
Source: mSphere. 2024 Mar 19;9(4):e00799-23. doi: 10.1128/msphere.00799-23 (PMC11036806; doi:10.1128/msphere.00799-23)
Supplement: Fig. S2 — LTag sequences aligned. [file msphere.00799-23-s0002.pdf]

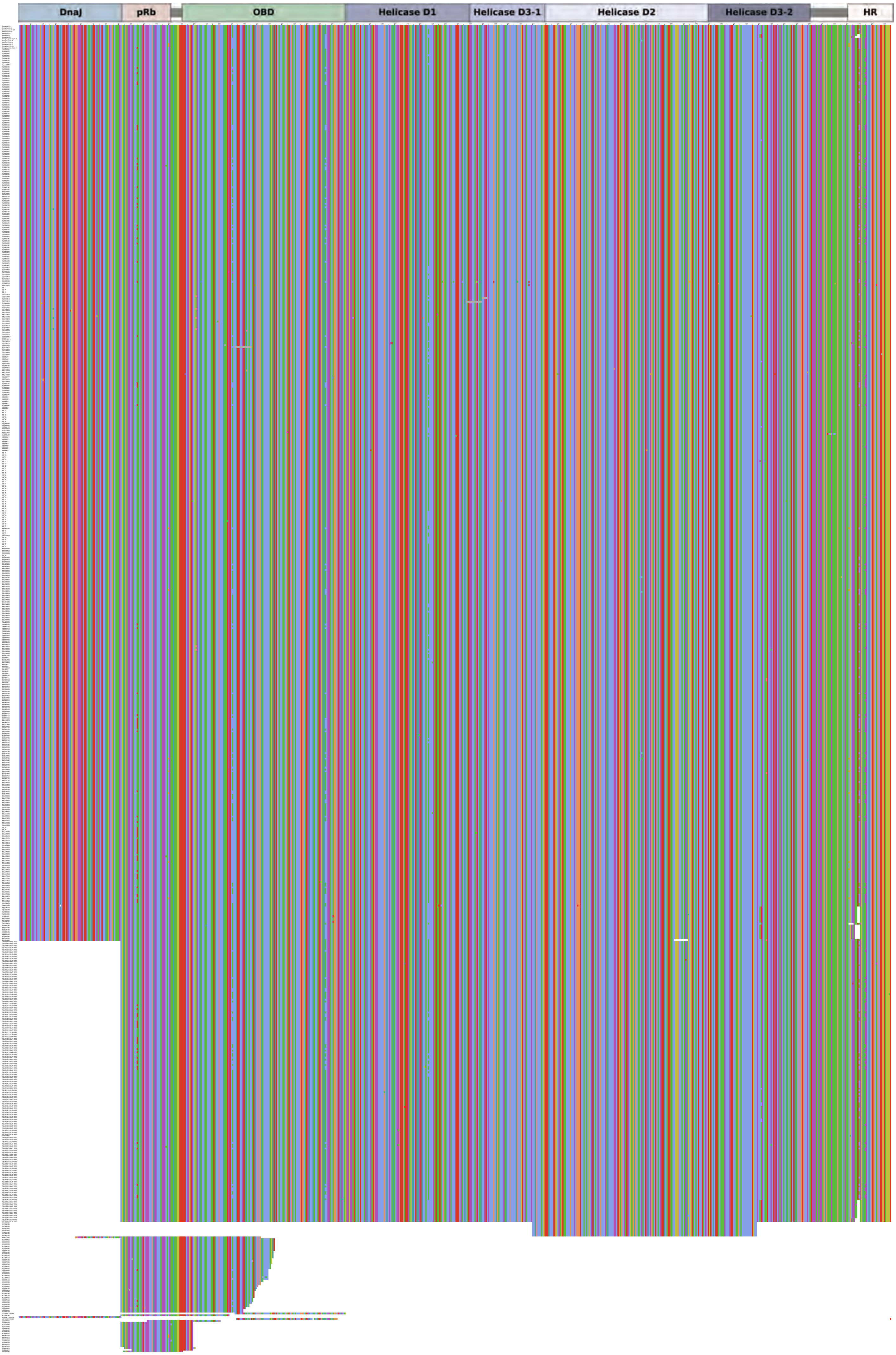
DnaJ
pRb
OBD
Helicase D1
Helicase D3-1
Helicase D2
Helicase D3-2
HR

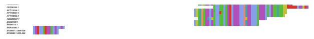

Category	Residues
Hydrophobic	A,I,L,M,F,W,V
Positive charge	K,R
Negative charge	E,D
Polar	N,Q,S,T
Cysteines	C
Glycines	G
Prolines	P
Aromatic	H,Y
Ambiguous	B,Z,J,X
No sequence/ gap	-

Supplementary Figure 2. LTag alignment.
Rendered in JalView [1] following the Clustal color scheme [2].

[1] Waterhouse, A.M., Procter, J.B., Martin, D.M.A, Clamp, M. and Barton, G. J. (2009) "Jalview Version 2 - a multiple sequence alignment editor and analysis workbench" Bioinformatics 25 (9) 1189-1191 doi: 10.1093/bioinformatics/btp033

[2] Larkin MA, Blackshields G, Brown NP, Chenna R, McGettigan PA, McWilliam H, Valentin F, Wallace IM, Wilm A, Lopez R, Thompson JD, Gibson TJ, Higgins DG.(2007). Clustal W and Clustal X version 2.0. Bioinformatics, 23, 2947-2948.
