# Supplementary material for: Structural implications of BK polyomavirus sequence variations in the major viral capsid protein Vp1 and large T-antigen: a computational study
Source: mSphere. 2024 Mar 19;9(4):e00799-23. doi: 10.1128/msphere.00799-23 (PMC11036806; doi:10.1128/msphere.00799-23)
Supplement: Fig. S3 — Vp1 conformational variability. [file msphere.00799-23-s0003.pdf]

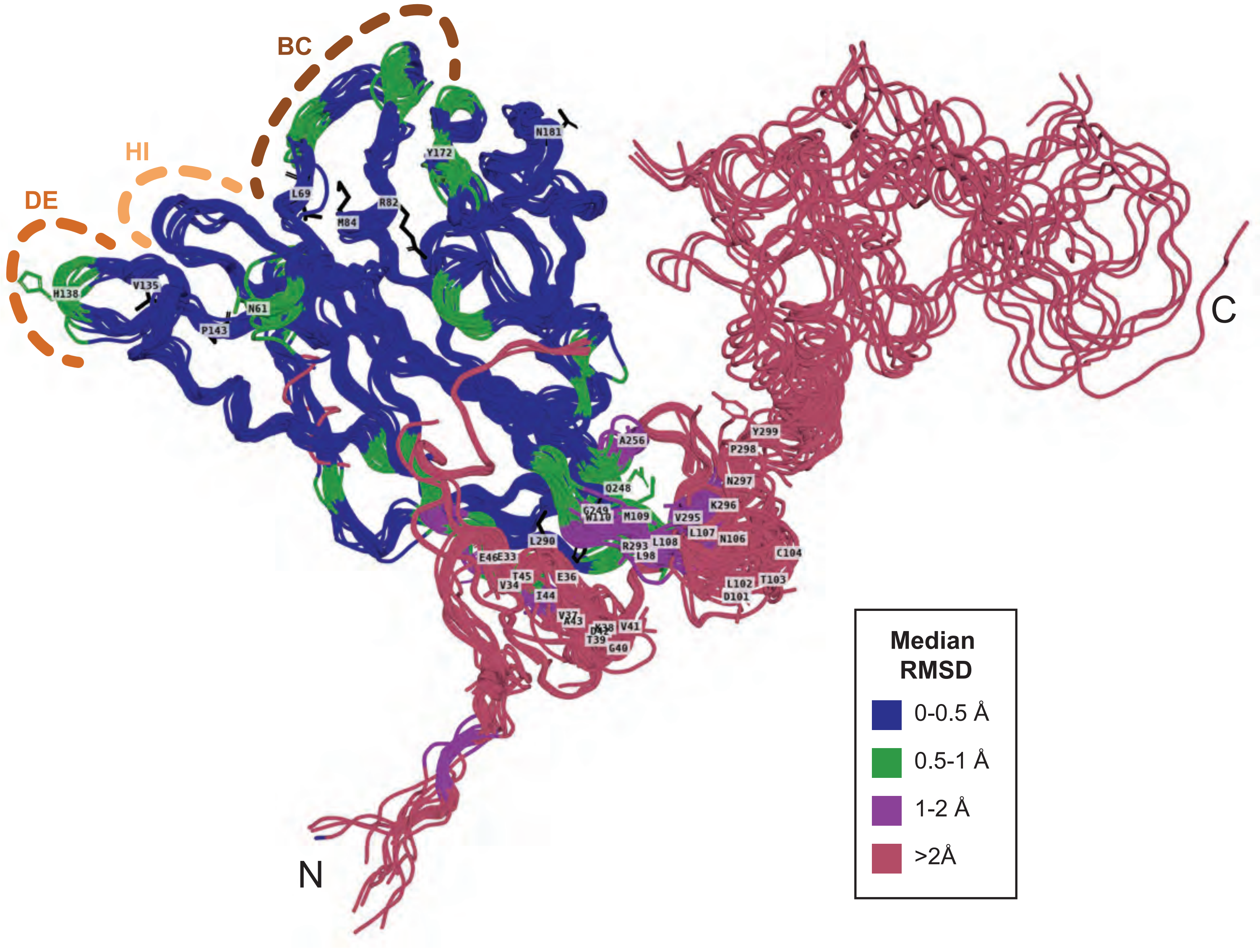

Supplementary Figure 3. Vp1 conformational differences across available structures. Superposition of all 58 chains of 9 Vp1 structures (PDB IDs: 7ZIQ, 6GG0, 6ESB, 7B6C, 7B6A, 4MJ1, 5FUA, 7B69, 4MJ0), colored by median Euclidean distance of corresponding Cα positions at each residue. The BC, HI and DE loops are indicated with dashed lines. Residues with low average per-residue median electron density support for individual atoms (EDIAm < 0.8) are labelled and shown as black sticks.
