# Supplementary material for: Structural implications of BK polyomavirus sequence variations in the major viral capsid protein Vp1 and large T-antigen: a computational study
Source: mSphere. 2024 Mar 19;9(4):e00799-23. doi: 10.1128/msphere.00799-23 (PMC11036806; doi:10.1128/msphere.00799-23)
Supplement: TABLE S1 [file msphere.00799-23-s0004.pdf]

Supplementary Table 1. Summary of Vp1 variant positions discussed in the text. GenBank accessions are listed for unpublished studies.

**BKPyV Vp1 AA residue number**	**BKPyV Serotype and AA** (rate%)	**JCPyV AA** (rate%)	**SV40 AA** (rate%)	**SXM** (count)	**SIM** (count)	**Patients** (number)	**Interactions**	**Annotations**	**Source** (counts)	**Country** (counts)	**Studies** (counts)	**Study IDs**
20	SI: E(86%) D(13%), SII: E, SIII: E, SIV: E(88%) D(12%)	D(98.8%), H(1.1%)	E			SI: A(5)	Intra-pentamer	Predicted BCR epitope	urine(5)	Switzerland(5)	1	[1]
60	D	D(97.7%), N(2.0%)	D		E(23), N(12), H(10), A(4), Y(2)		Sialic acid, Antibody [50], [51], [52]	Predicted BCR epitope, APOBEC3	urine(27), organ(17), blood(3)	Iran(21), USA(6), France(3), Other(21)	15	[2], [3], [4], [GenBank: JN793992.1 - JN794043.1], [5], [6], [7], [8], [GenBank: LC606235.1 - LC606249.1], [6], [9], [10], [GenBank: MG871470.1 - MG871556.1], [11], [12]
61	SI: E(98%), SII: E(50%) N(3%), SIII: D(73%) E(9%), SIV: N(75%)	E(99.9%), G(0.1%)	E	SI: D(2) N(1), SII: D(57), SIV: E(1)	S(69), Q(5), T(4), K(1)		Intra-pentamer, Antibody [50 51, 52]	Predicted BCR epitope, Low electron density support, APOBEC3	urine(96), blood(14), organ(6), water(1)	China(11), Thailand(10), Germany(8), Other(111)	36	[2], [4], [GenBank: JN793992.1 - JN794043.1], [5], [13], [6], [18], [14], [10], [15], [16], [GenBank: MW678842.1 - MW678843.1], [11], [GenBank: KF740806.1 - KF740824.1], [17], [GenBank: KF443800.1, KY284052.1 - KY284057.1, KY560239.1 - KY560244.1], [Genbank: MK122953.1 - MK122963.1], [19], [7], [6], [20], [21], [22], [23], [24], [25], [26], [27], [GenBank: MG871470.1 - MG871556.1], [Genbank: MF817711.1 - MF817712.1], [21], [28], [29], [30], [31], [32]
62	SI: N(98%), SII: N(95%) D(3%), SIII: H(45%) N(36%), SIV: D(96%)	H(99.8%), N(0.2%)	H	SI: H(6) D(2), SIV: N(8) H(2)	V(4), S(2), K(2), T(1), E(1)		Intra-pentamer, Antibody [52]	Predicted BCR epitope, APOBEC3	urine(16), blood(3), organ(3)	Brazil(4), USA(3), Kuwait(3), Other(18)	13	[2], [19], [7], [21], [33], [34], [GenBank: MG871470.1 - MG871556.1], [21], [17], [29], [19], [31], [32]

68	SI: L(96%), SII: L, SIII: Q, SIV: L(99%)	K(98.7%), N(0.9%)	K	SI: Q(1), SIV: Q(2)	V(27)		Sialic acid, Antibody [51]		urine(15), organ(14)	Iran(15), Thailand(3), Brazil(3), Other(9)	7	[3], [26], [4], [GenBank: MG871470.1 - MG871556.1], [12], [21], [30]
69	SI: K(97%), SII: K, SIII: H, SIV: R(96%)	S(98.4%), P(1.3%)	S	SI: H(1) R(9), SIV: K(12)	Q(1), E(1), M(1), N(1)		Sialic acid, Antibody [51, 52]	Low electron density support, APOBEC3	urine(16), organ(2)	Japan(4), Spain(3), China(3), Other(16)	13	[2], [4], [GenBank: JN793992.1 - JN794043.1], [6], [8], [33], [10], [26], [16], [17], [29], [12], [31]
71	SI: S(99%), SII: T(53%), SIII: T(55%) S(45%), SIV: T(99%)	S(99.9%), A(0.1%)	A	SI: T(2), SII: S(58), SIV: S(1)	I(2)		Intra-pentamer, Antibody		saliva(58), urine(3), blood(1), organ(1)	Brazil(58), Kuwait(2), Japan(2), Other(1)	5	[2], [12], [GenBank: OM628999.1 - OM629056.1], [GenBank: KY114802.1 - KY114803.1], [19]
72	A	I(99.8%), T(0.1%)	A		V(37), G(2), S(2), T(1)		Intra-pentamer, Antibody* [51, 52]		urine(21), organ(17)	Iran(15), Brazil(5), USA(4), Other(18)	13	[3], [2], [4], [12], [35], [36], [21], [GenBank: KC412415.1 - KC412484.1], [27], [GenBank: MG871470.1 - MG871556.1], [30], [31], [37]
73	SI: E(93%) K(1%), SII: E, SIII: E, SIV: E(97%)	S	E	SIV: K(2)	A(26), Q(24), G(5), I(1)		Intra-pentamer, Antibody* [51, 52]	APOBEC3	urine(32), organ(17), blood(1)	Iran(15), USA(6), Brazil(6), Other(31)	22	[2], [4], [38], [GenBank: JN793992.1 - JN794043.1], [13], [10], [12], [18], [3], [6], [8], [21], [GenBank: KC412415.1 - KC412484.1], [23], [GenBank: HE650855.1, HE650859.1, HE650860.1, HE650864.1, HE650866.1, HE650877.1, HE650878.1, HE652114.1], [34], [GenBank: MG871470.1 - MG871556.1], [Genbank: MF817711.1 - MF817712.1], [29], [30], [31], [37]
75	SI: D(97%), SII: A(98%), SIII: A, SIV: A(99%)	T(99.8%), P(0.1%)	Q	SII: D(3), SIV: D(2)	N(18), E(1), H(1), T(1)	SI: N(3)	Intra-pentamer, Antibody	Predicted BCR epitope, APOBEC3	urine(18), blood(2), organ(1)	USA(4), Spain(3), Brazil(3), Other(19)	16	[2], [4], [5], [1], [GenBank: LC029411.1 - LC029414.1], [Genbank: MF817711.1 - MF817712.1], [6], [GenBank: KC412415.1 - KC412484.1], [16], [GenBank: MG871470.1 - MG871556.1], [36], [GenBank: LC029411.1 - LC029414.1], [11], [17], [31], [18]

77	SI: S(98%), SII: D(97%), SIII: E(50%) D(50%), SIV: D(71%) E(21%) N(3%)	E(95.9%), D(2.0%)	T	SI: N(4) D(1), SII: N(1)	Q(9), H(8), K(1)		Antibody [51, 52]	Predicted BCR epitope, APOBEC3	urine(18)	France(3), Japan(3), India(2), Other(16)	12	[29], [4], [GenBank: JN793992.1 - JN794043.1], [5], [6], [GenBank: MG871470.1 - MG871556.1], [11], [17], [GenBank: KF443800.1, KY284052.1 - KY284057.1, KY560239.1 - KY560244.1], [12], [31], [37]
82	SI: E(88%) D(7%), SII: D(98%), SIII: D, SIV: D(98%)	N(82.2%), S(17.2%)	D(92.3 %), E(7.7%)	SII: E(3), SIV: E(3)	Q(31), K(3), N(3), Y(1), G(1)	SI: Q(5)	Antibody [51, 52]	Predicted BCR epitope, Low electron density support, APOBEC3	urine(24), organ(17), blood(1)	Iran(17), Japan(7), Switzerland(5), Other(21)	17	[3], [19], [4], [2], [1], [35], [36], [13], [6], [GenBank: HE650855.1, HE650859.1, HE650860.1, HE650864.1, HE650866.1, HE650877.1, HE650878.1, HE652114.1], [25], [16], [GenBank: MG871470.1 - MG871556.1], [17], [29], [12], [31]
83	SI: R(97%), SII: K(50%), SIII: K(59%) R(41%), SIV: R(99%)	K(96.4%), R(3.6%)	K	SI: K(18), SII: R(61), SIV: K(2)	E(1)		Antibody [50]	APOBEC3	saliva(58), urine(21), organ(1)	Brazil(59), Japan(8), Iran(5), Other(10)	16	[2], [19], [4], [12], [5], [6], [35], [36], [8], [GenBank: KC412415.1 - KC412484.1], [30], [24], [25], [GenBank: OM628999.1 - OM629056.1], [GenBank: AB443953.1 - AB444045.1], [GenBank: LC169110.1 - LC169116.1, LC164373.1- LC164386.1]
116	V	L(99.6%), V(0.3%)	V		S(5), A(4), D(3), T(2), L(1), N(1), E(1)				urine(14)	Iran(11), Brazil(3), Spain(1), Other(2)	6	[19], [GenBank: LC606235.1 - LC606249.1], [31], [39], [GenBank: KC412415.1 - KC412484.1], [33]
117	SI: Q(98%), SII: K(97%), SIII: K, SIV: K(99%)	K(99.9%), R(0.1%)	K	SI: K(9), SII: Q(3), SIV: Q(1)	N(4), R(1), T(1)		Vp2/Vp3		urine(15), blood(1)	Iran(10), Sri Lanka(3), Germany(2), Other(4)	9	[GenBank: LC606235.1 - LC606249.1], [GenBank: KR075893.1 - KR075908.1], [30], [GenBank: LC169110.1 - LC169116.1, LC164373.1- LC164386.1], [GenBank: KY114802.1 - KY114803.1], [11], [17], [29], [GenBank: AY683133.1]
119	E	E(99.9%), Q(0.1%)	E		S(5), R(3), V(3), G(2), K(2), W(1)			APOBEC3	urine(13), saliva(2), water(1)	Iran(13), Brazil(2), Portugal(1)	3	[GenBank: LC606235.1 - LC606249.1], [GenBank: LC169110.1 - LC169116.1, LC164373.1- LC164386.1], [GenBank: OM628999.1 - OM629056.1]

120	V	V(99.8%), R(0.1%)	V		S(11), R(3), Q(2), K(1), F(1), Y(1), T(1)				urine(20)	Iran(20)	2	[GenBank: LC606235.1 - LC606249.1], [GenBank: LC169110.1 - LC169116.1, LC164373.1- LC164386.1]
121	SI: I(97%), SII: I(98%), SIII: I, SIV: I(90%) M(7%)	I(84.4%), L(15.5%)	I	SI: M(2), SII: M(2)	V(4), T(3), R(3), D(2), L(2), P(2), N(1), E(1), S(1), C(1)		Intra-pentamer		urine(23), organ(1)	Iran(18), Japan(3), Finland(2), Other(1)	6	[GenBank: LC606235.1 - LC606249.1], [38], [12], [40], [GenBank: LC169110.1 - LC169116.1, LC164373.1- LC164386.1], [33]
139	SI: H(97%), SII: N(98%), SIII: N, SIV: N(100%)	N(99.6%), K(0.3%)	N	SI: N(13)	Q(1), D(1), T(1), K(1)		Intra-pentamer, Sialic acid	Predicted BCR epitope	urine(9), saliva(1)	Brazil(5), USA(4), France(2), Other(6)	7	[4], [GenBank: JN793992.1 - JN794043.1], [GenBank: OM628999.1 - OM629056.1], [39], [17], [GenBank: KC412415.1 - KC412484.1], [37]
167	M	F(99.9%), C(0.1%)	A		L(34)		Intra-pentamer		urine(18), organ(13), blood(2)	Kuwait(13), Thailand(8), Sri Lanka(2), Other(11)	9	[2], [41], [5], [42], [43], [GenBank: MG871470.1 - MG871556.1], [11], [GenBank: KF443800.1, KY284052.1 - KY284057.1, KY560239.1 - KY560244.1], [GenBank: HE650855.1, HE650859.1, HE650860.1, HE650864.1, HE650866.1, HE650877.1, HE650878.1, HE652114.1]
172	K	K(77.4%), T(21.0%)	K		S(4), R(4), N(2)		Antibody [51]	Predicted BCR epitope, Low electron density support	organ(9)	Kuwait(9), Iran(1)	4	[2], [GenBank: HE650855.1, HE650859.1, HE650860.1, HE650864.1, HE650866.1, HE650877.1, HE650878.1, HE652114.1], [43], [GenBank: KX816853.1 - KX816854.1]
175	SI: E(51%) D(47%), SII: Q(92%), SIII: Q, SIV: E(96%)	D(99.6%), E(0.2%)	A	SI: Q(1), SIV: D(6) Q(1)	H(4)		Antibody [50, 51]	Predicted BCR epitope, APOBEC3	urine(10), blood(1), water(1)	India(4), Sri Lanka(3), USA(1), Other(4)	6	[4], [44], [45], [GenBank: MG871470.1 - MG871556.1], [11], [GenBank: KF443800.1, KY284052.1 - KY284057.1, KY560239.1 - KY560244.1]
178	SI: I(90%), SII: I, SIII: I, SIV: V	I	V	SI: V(45)		SI: V(14)	Antibody	Predicted BCR epitope	urine(36), water(2), blood(1)	Brazil(28), Switzerland(14), USA(7), Other(10)	7	[46], [4], [1], [47], [39], [GenBank: KC412415.1 - KC412484.1], [10]
349	R	R(99.9%), K(0.1%)	R			SI: S(1)			urine(1)	Switzerland(1)	1	[1]
353	SI: K(98%), SII: R, SIII: R, SIV: R	R(88.9%), K(10.8%)	E	SI: R(2)		SI: R(1)			organ(1), urine(1)	USA(1), Switzerland(1), Other(1)	3	[38], [1], [14]

362	SI: L(87%), SII: V, SIII: V(93%), SIV: V(98%)	L(99.4%), M(0.3%)	Q	SI: V(67), SIII: L(1), SIV: L(3)					urine(47), blood(11), water(2), organ(2)	Belgium(13), USA(13), Germany(11), Other(34)	15	[4], [38], [GenBank: JN793992.1 - JN794043.1], [5], [35], [40], [14], [10], [27], [48], [16], [49], [47], [28], [30]

* Antibody interactions inferred from mutational analysis with neutralisation assays, structural evidence of antibody binding from experimentally solved structures is not currently available

[1] K. Leuzinger *et al.*, “Molecular Characterization of BK Polyomavirus Replication in Allogeneic Hematopoietic Cell Transplantation Patients,” *J. Infect. Dis.*, vol. 227, no. 7, pp. 888–900, Apr. 2023, doi: 10.1093/infdis/jiac450.

[2] W. Chehadeh and M. R. Nampoory, “Genotypic diversity of polyomaviruses circulating among kidney transplant recipients in Kuwait,” *J. Med. Virol.*, vol. 85, no. 9, pp. 1624–1631, Sep. 2013, doi: 10.1002/jmv.23639.

[3] M. Vaezjalali, H. Azimi, S. M. Hosseini, A. Taghavi, and H. Goudarzi, “Different Strains of BK Polyomavirus: VP1 Sequences in a Group of Iranian Prostate Cancer Patients,” *Urol. J.*, vol. 15, no. 2, pp. 44–48, Mar. 2018, doi: 10.22037/uj.v0i0.3833.

[4] A. Addetia *et al.*, “In Vivo Generation of BK and JC Polyomavirus Defective Viral Genomes in Human Urine Samples Associated with Higher Viral Loads,” *J. Virol.*, vol. 95, no. 12, pp. e00250-21, May 2021, doi: 10.1128/JVI.00250-21.

[5] H.-Y. Zheng *et al.*, “Relationships between BK virus lineages and human populations,” *Microbes Infect.*, vol. 9, no. 2, pp. 204–213, Feb. 2007, doi: 10.1016/j.micinf.2006.11.008.

[6] Q. Chen *et al.*, “Subtype IV of the BK polyomavirus is prevalent in East Asia,” *Arch. Virol.*, vol. 151, no. 12, pp. 2419–2429, Dec. 2006, doi: 10.1007/s00705-006-0814-z.

[7] B. Kapusinszky *et al.*, “BK polyomavirus subtype III in a pediatric renal transplant patient with nephropathy,” *J. Clin. Microbiol.*, vol. 51, no. 12, pp. 4255–4258, Dec. 2013, doi: 10.1128/JCM.01801-13.

[8] S. Zhong *et al.*, “Comparison of the distribution patterns of BK polyomavirus lineages among China, Korea and Japan: implications for human migrations in northeast Asia,” *Microbiol. Immunol.*, vol. 53, no. 5, pp. 266–276, May 2009, doi: 10.1111/j.1348-0421.2009.00121.x.

[9] A. Touzé, L. Bousarghin, C. Ster, A.-L. Combita, P. Roingeard, and P. Coursaget, “Gene transfer using human polyomavirus BK virus-like particles expressed in insect cells,” *J. Gen. Virol.*, vol. 82, no. Pt 12, pp. 3005–3009, Dec. 2001, doi: 10.1099/0022-1317-82-12-3005.

[10] O. Mineeva-Sangwo *et al.*, “Polyomavirus BK Genome Comparison Shows High Genetic Diversity in Kidney Transplant Recipients Three Months after Transplantation,” *Viruses*, vol. 14, no. 7, p. 1533, Jul. 2022, doi: 10.3390/v14071533.

[11] A. K. D. V. Yashodha Ratnayake, N. Fernando, T. Gajanayake, S. M. Handunnetti, and S. C. Jude Jayamaha, “Molecular characterization of BK virus detected in renal transplant patients in Sri Lanka: a preliminary study,” *Indian J. Med. Res.*, vol. 156, no. 3, pp. 500–507, Sep. 2022, doi: 10.4103/ijmr.IJMR_79_20.

[12] S. Zhong *et al.*, “Even distribution of BK polyomavirus subtypes and subgroups in the Japanese Archipelago,” *Arch. Virol.*, vol. 152, no. 9, pp. 1613–1621, 2007, doi: 10.1007/s00705-007-0997-y.

[13] H. Boukoum *et al.*, “Distribution of BK polyomavirus genotypes in Tunisian renal transplant recipients,” *J. Med. Virol.*, vol. 83, no. 4, pp. 725–730, Apr. 2011, doi: 10.1002/jmv.22035.

[14] L. Jin, P. E. Gibson, W. A. Knowles, and J. P. Clewley, “BK virus antigenic variants: sequence analysis within the capsid VP1 epitope,” *J. Med. Virol.*, vol. 39, no. 1, pp. 50–56, Jan. 1993, doi: 10.1002/jmv.1890390110.

[15] J. Sachithanandham *et al.*, “Detection of opportunistic DNA viral infections by multiplex PCR among HIV infected individuals receiving care at a tertiary care hospital in South India,” *Indian J. Med. Microbiol.*, vol. 27, no. 3, pp. 210–216, 2009, doi: 10.4103/0255-0857.53202.

[16] A. Bárcena-Panero, M. Van Ghelue, M. T. H. Khan, J. E. Echevarría, G. Fedele, and U. Moens, "BK virus-associated infection in cerebrospinal fluid of neurological patients and mutation analysis of the complete VP1 gene in different patient groups," *J. Cell. Physiol.*, vol. 227, no. 1, pp. 136–145, Jan. 2012, doi: 10.1002/jcp.22711.

[17] S. Akhgari *et al.*, "Frequency and subtype of BK virus infection in Iranian patients infected with HIV," *Med. Microbiol. Immunol. (Berl.)*, vol. 205, no. 1, pp. 57–62, Feb. 2016, doi: 10.1007/s00430-015-0426-x.

[18] P. Q. Toan *et al.*, "Identification of BK Virus Genotypes in Recipients of Renal Transplant in Vietnam," *Transplant. Proc.*, vol. 51, no. 8, pp. 2683–2688, Oct. 2019, doi: 10.1016/j.transproceed.2019.03.072.

[19] S. Zhong *et al.*, "Age-related urinary excretion of BK polyomavirus by nonimmunocompromised individuals," *J. Clin. Microbiol.*, vol. 45, no. 1, pp. 193–198, Jan. 2007, doi: 10.1128/JCM.01645-06.

[20] R. M. Lonergan *et al.*, "Reactivation of BK polyomavirus in patients with multiple sclerosis receiving natalizumab therapy," *J. Neurovirol.*, vol. 15, no. 5–6, pp. 351–359, Sep. 2009, doi: 10.3109/13550280903131923.

[21] J. Comerlato *et al.*, "Molecular detection and characterization of BK and JC polyomaviruses in urine samples of renal transplant patients in Southern Brazil," *J. Med. Virol.*, vol. 87, no. 3, pp. 522–528, Mar. 2015, doi: 10.1002/jmv.24086.

[22] A. Krumbholz, P. Wutzler, and R. Zell, "The non-coding region of BK subtype II viruses," *Virus Genes*, vol. 36, no. 1, pp. 27–29, Feb. 2008, doi: 10.1007/s11262-007-0166-0.

[23] M. J. Carr, G. P. McCormack, K. J. Mutton, and B. Crowley, "Unique BK virus non-coding control region (NCCR) variants in hematopoietic stem cell transplant recipients with and without hemorrhagic cystitis," *J. Med. Virol.*, vol. 78, no. 4, pp. 485–493, Apr. 2006, doi: 10.1002/jmv.20566.

[24] S. Bofill-Mas, M. Formiga-Cruz, P. Clemente-Casares, F. Calafell, and R. Girones, "Potential transmission of human polyomaviruses through the gastrointestinal tract after exposure to virions or viral DNA," *J. Virol.*, vol. 75, no. 21, pp. 10290–10299, Nov. 2001, doi: 10.1128/JVI.75.21.10290-10299.2001.

[25] T. Takasaka *et al.*, "Stability of the BK polyomavirus genome in renal-transplant patients without nephropathy," *J. Gen. Virol.*, vol. 87, no. Pt 2, pp. 303–306, Feb. 2006, doi: 10.1099/vir.0.81368-0.

[26] A. Krumbholz *et al.*, "Prevalence of BK virus subtype I in Germany," *J. Med. Virol.*, vol. 78, no. 12, pp. 1588–1598, Dec. 2006, doi: 10.1002/jmv.20743.

[27] Y. Yogo, S. Zhong, M. Suzuki, A. Shibuya, and T. Kitamura, "Occurrence of the European subgroup of subtype I BK polyomavirus in Japanese-Americans suggests transmission outside the family," *J. Virol.*, vol. 81, no. 23, pp. 13254–13258, Dec. 2007, doi: 10.1128/JVI.01018-07.

[28] C. Torres *et al.*, "High diversity of human polyomaviruses in environmental and clinical samples in Argentina: Detection of JC, BK, Merkel-cell, Malawi, and human 6 and 7 polyomaviruses," *Sci. Total Environ.*, vol. 542, no. Pt A, pp. 192–202, Jan. 2016, doi: 10.1016/j.scitotenv.2015.10.047.

[29] C. Hu, Y. Huang, J. Su, M. Wang, Q. Zhou, and B. Zhu, "The prevalence and isolated subtypes of BK polyomavirus reactivation among patients infected with human immunodeficiency virus-1 in southeastern China," *Arch. Virol.*, vol. 163, no. 6, pp. 1463–1468, Jun. 2018, doi: 10.1007/s00705-018-3724-y.

[30] C. Schmitt, L. Raggub, S. Linnenweber-Held, O. Adams, A. Schwarz, and A. Heim, "Donor origin of BKV replication after kidney transplantation," *J. Clin. Virol. Off. Publ. Pan Am. Soc. Clin. Virol.*, vol. 59, no. 2, pp. 120–125, Feb. 2014, doi: 10.1016/j.jcv.2013.11.009.

[31] J. Ledesma, E. Bouza, M. A. González-Nicolás, D. García de Viedma, B. Rodríguez-Sánchez, and P. Muñoz, "BK polyomavirus genotyping at inter- and intra-patient level in Spain," *J. Med. Virol.*, vol. 85, no. 8, pp. 1402–1408, Aug. 2013, doi: 10.1002/jmv.23612.

[32] B. K. Saundh, R. Baker, M. Harris, M. P. Welberry Smith, A. Cherukuri, and A. Hale, "Early BK polyomavirus (BKV) reactivation in donor kidney is a risk factor for development of BKV-associated nephropathy," *J. Infect. Dis.*, vol. 207, no. 1, pp. 137–141, Jan. 2013, doi: 10.1093/infdis/jis642.

[33] H. Ikegaya *et al.*, "Identification of a genomic subgroup of BK polyomavirus spread in European populations," *J. Gen. Virol.*, vol. 87, no. Pt 11, pp. 3201–3208, Nov. 2006, doi: 10.1099/vir.0.82266-0.

[34] S. Slavov, I. Tsekov, and Z. Kalvatchev, "Sequence variations of the VP1 gene of Polyomavirus hominis 1 among Bulgarians," *J. Med. Virol.*, vol. 82, no. 2, pp. 325–330, Feb. 2010, doi: 10.1002/jmv.21684.

[35] Y. Nishimoto *et al.*, "Evolution of BK virus based on complete genome data," *J. Mol. Evol.*, vol. 63, no. 3, pp. 341–352, Sep. 2006, doi: 10.1007/s00239-005-0092-5.
[36] T. Takasaka *et al.*, "Subtypes of BK virus prevalent in Japan and variation in their transcriptional control region," *J. Gen. Virol.*, vol. 85, no. Pt 10, pp. 2821–2827, Oct. 2004, doi: 10.1099/vir.0.80363-0.
[37] E. Krautkrämer, T. M. Klein, C. Sommerer, P. Schnitzler, and M. Zeier, "Mutations in the BC-loop of the BKV VP1 region do not influence viral load in renal transplant patients," *J. Med. Virol.*, vol. 81, no. 1, pp. 75–81, Jan. 2009, doi: 10.1002/jmv.21359.
[38] Y. Chen, P. M. Sharp, M. Fowkes, O. Kocher, J. T. Joseph, and I. J. Koralnik, "Analysis of 15 novel full-length BK virus sequences from three individuals: evidence of a high intra-strain genetic diversity," *J. Gen. Virol.*, vol. 85, no. Pt 9, pp. 2651–2663, Sep. 2004, doi: 10.1099/vir.0.79920-0.
[39] P. R. P. Urbano *et al.*, "Variable sources of Bk virus in renal allograft recipients," *J. Med. Virol.*, vol. 91, no. 6, pp. 1136–1141, Jun. 2019, doi: 10.1002/jmv.25409.
[40] H. Hosoi *et al.*, "A cluster of BK polyomavirus-associated hemorrhagic cystitis after allogeneic hematopoietic stem cell transplantation," *Transpl. Infect. Dis. Off. J. Transplant. Soc.*, vol. 23, no. 6, p. e13736, Dec. 2021, doi: 10.1111/tid.13736.
[41] A. Jenkins, S. Govind, C. Morris, and N. Berry, "Complete Genome Sequence of Original Material Used To Derive the WHO International Standard for Human Polyomavirus BK DNA," *Microbiol. Resour. Announc.*, vol. 8, no. 43, pp. e00911-19, Oct. 2019, doi: 10.1128/MRA.00911-19.
[42] R. J. Drew, A. Walsh, B. N. Laoi, and B. Crowley, "Phylogenetic analysis of the complete genome of 11 BKV isolates obtained from allogenic stem cell transplant recipients in Ireland," *J. Med. Virol.*, vol. 84, no. 7, pp. 1037–1048, Jul. 2012, doi: 10.1002/jmv.23240.
[43] W. Chehadeh, S. S. Kurien, and M. R. Nampoory, "Molecular characterization of BK and JC viruses circulating among potential kidney donors in Kuwait," *BioMed Res. Int.*, vol. 2013, p. 683464, 2013, doi: 10.1155/2013/683464.
[44] S. Bofill-Mas, S. Pina, and R. Girones, "Documenting the epidemiologic patterns of polyomaviruses in human populations by studying their presence in urban sewage," *Appl. Environ. Microbiol.*, vol. 66, no. 1, pp. 238–245, Jan. 2000, doi: 10.1128/AEM.66.1.238-245.2000.
[45] S. Bendiksen, O. P. Rekvig, M. Van Ghelue, and U. Moens, "VP1 DNA sequences of JC and BK viruses detected in urine of systemic lupus erythematosus patients reveal no differences from strains expressed in normal individuals," *J. Gen. Virol.*, vol. 81, no. Pt 11, pp. 2625–2633, Nov. 2000, doi: 10.1099/0022-1317-81-11-2625.
[46] D. J. Kenan, P. A. Mieczkowski, E. Latulippe, I. Côté, H. K. Singh, and V. Nickeleit, "BK Polyomavirus Genomic Integration and Large T Antigen Expression: Evolving Paradigms in Human Oncogenesis," *Am. J. Transplant. Off. J. Am. Soc. Transplant. Am. Soc. Transpl. Surg.*, vol. 17, no. 6, pp. 1674–1680, Jun. 2017, doi: 10.1111/ajt.14191.
[47] P. M. Sharma, G. Gupta, A. Vats, R. Shapiro, and P. Randhawa, "Phylogenetic analysis of polyomavirus BK sequences," *J. Virol.*, vol. 80, no. 18, pp. 8869–8879, Sep. 2006, doi: 10.1128/JVI.00510-06.
[48] C. Sugimoto, K. Hara, F. Taguchi, and Y. Yogo, "Regulatory DNA sequence conserved in the course of BK virus evolution," *J. Mol. Evol.*, vol. 31, no. 6, pp. 485–492, Dec. 1990, doi: 10.1007/BF02102075.
[49] K. T. Momynaliev, E. V. Gorbatenko, A. B. Shevtsov, O. G. Gribanov, N. N. Babenko, and M. M. Kaabak, "Prevalence and subtypes of BK virus in pediatric renal transplant recipients in Russia," *Pediatr. Transplant.*, vol. 16, no. 2, pp. 151–159, Mar. 2012, doi: 10.1111/j.1399-3046.2011.01640.x.
[50] J. M. Lindner *et al.*, "Human Memory B Cells Harbor Diverse Cross-Neutralizing Antibodies against BK and JC Polyomaviruses," *Immunity*, vol. 50, no. 3, pp. 668-676.e5, Mar. 2019, doi: 10.1016/j.immuni.2019.02.003.
[51] N.-K. Nguyen *et al.*, "A cluster of broadly neutralizing IgG against BK polyomavirus in a repertoire dominated by IgM," *Life Sci. Alliance*, vol. 6, no. 4, p. e202201567, Apr. 2023, doi: 10.26508/lsa.202201567.
[52] D. McIlroy *et al.*, "Persistent BK Polyomavirus Viruria is Associated with Accumulation of VP1 Mutations and Neutralization Escape," *Viruses*, vol. 12, no. 8, p. 824, Jul. 2020, doi: 10.3390/v12080824.
